# The effect of metabolic syndrome on male reproductive health: A cross-sectional study in a group of fertile men and male partners of infertile couples

**DOI:** 10.1371/journal.pone.0194395

**Published:** 2018-03-16

**Authors:** Kristel Ehala-Aleksejev, Margus Punab

**Affiliations:** Andrology Centre, Tartu University Hospital, Tartu, Estonia; Universite Clermont Auvergne, FRANCE

## Abstract

This study aimed to determine the effect of metabolic syndrome (MS) on the reproductive function in fertile (FM) and male partners of infertile couples (MPIC). We performed a cross-sectional study formatting two study groups: partners of pregnant women (n = 238; mean age 32.0) as FM and male partners of infertile couples (n = 2642; mean age 32.6) as MPIC. A standard semen analysis was performed and clinical, laboratory and lifestyle data were analysed. The adapted NCEP-ATPIII criteria were used to define MS. 12.2% of FM and 17.8% of MPIC had MS. In both groups, men with MS were older, they were centrally obese and had higher triglycerides, systolic and diastolic blood pressure and decreased HDL cholesterol values as compared to men without MS. However, glucose concentrations as well as fasting insulin levels were significantly higher only in the MPIC-MS^+^ group. MS was not associated with semen parameters. Testosterone levels were negatively correlated to MS in both groups. This negative association persisted within the BMI categories between MPIC-MS^-^ and MPIC-MS^+^ groups. LH was negatively correlated to MS but only in MPIC. FSH and oestradiol were not correlated to MS. Smoking and alcohol consumption were higher among men with MS. This study shows that except for testosterone, MS has no independent effect on major fertility parameters in different subgroups of men.

## Introduction

Metabolic syndrome (MS) is a complex of medical conditions characterized by abdominal obesity, dyslipidemia, hypertension and high fasting glucose. The worldwide prevalence of MS emerged not only as a predictor of cardiovascular disease but also as a potential contributing factor to male infertility. Although a link between MS and lower general health status has been proved [1–3], an association between male infertility and MS is yet under discussion.

There is strong evidence that MS is negatively associated with testosterone levels [2–5]] and might be positively related with plasma concentration of oestrogen [6]. Even if MS and the testosterone deficiency in men are closely linked then the effect of MS on male semen quality has not been sufficiently investigated on the basis of available data. There are some studies which have concluded that MS may affect male semen parameters [2, 4, 7, 8] and some which deny it [3, 9].

The purpose of this study was to investigate the eventual role of MS in male infertility assessing its impact on semen and hormonal parameters in a group of fertile men and male partners of infertile couples (MPIC).

## Methods

### Study design

This cross-sectional study was performed at the Andrology Centres of Tartu University Hospital in Tartu and in Tallinn with the service area covering the whole Estonia. Two study groups were formatted: partners of pregnant women as the fertile men's group (FM) and male partners of couples failing to conceive a child over a period of ≥12 months as MPIC.

### Partners of pregnant women

During 2010–2011, 3175 pregnant women who presented for prenatal care at Tartu University Women’s Clinic and West-Tallinn Central Hospital Women’s Clinic got informed about this study and their partners were invited to participate. The participants had a choice to complete only a questionnaire or in addition to filling out a questionnaire, to also pass a physical examination and give blood tests and/or semen analysis. Approximately 30% of the eligible men agreed to participate. Men were excluded from this study group because pregnancy was achieved by IVF, their abstinence time was too short or too long according to WHO guidelines [10], they had cryptorchidism, a history of surgery for cryptorchidism or for testicular cancer and they had urinary tract infections treated with antibiotics. Exclusion criteria also included missing information on body composition and blood tests. Accordingly, a total of 238 subjects were included in the study. The description of the study group and inclusion–exclusion criteria for participants has been described in detail elsewhere [11]

### Male partners of infertile couples

During 2008–2013, male partners of couples failing to conceive a child who presented for andrological examinations at the Tartu University Andrology Centres (Tartu and Tallinn) got informed about this study. They were invited to participate irrespective of their semen analysis results. In addition to the semen analysis, the study subjects went through a structured medical interview and were subjected to physical examinations and blood tests. They also had a choice to complete a questionnaire in addition to performed tests. The exclusion criteria included all cases of aspermia and azoospermia but also cases with absolute and severe causal factors of male infertility (fertility-related genetic problems, hypogonadotrophic hypogonadidm, cryptorchidism or other problems of genital development, seminal tract obstruction, history of testicular cancer or another tumor treated with chemo- or radiotherapy, abusing anabolic steroids or other medications with clear influence to spermatogenesis), men over age 50, an abstinence time too short or too long according to WHO guidelines [10], history of fertility problems of less than 1 year, missing information on body composition parameters and blood tests. Inclusion and exclusion criteria for participants have been described in detail elsewhere [12].

A total of 2642 (42.0%) of the 6286 males screened met the eligibility criteria.

The study was approved by the Ethics Committee on Human Research of the University of Tartu and written informed consent was obtained from all participants.

### Clinical examination

Testicular volumes were measured using the orchidometer (made of birch wood, Pharmacia & Upjohn, Denmark) and expressed in ml. The total testes volume (TTV) was the sum of right and left testicles. Disorders affecting testicular function [i.e. varicocele (gr 2, 3 and bilateral), testicular trauma, inguinal hernia] were recorded. Height was measured by roll-up metal length measuring tape for wall mounting, rounded to the nearest 0.1cm and expressed in cm, respectively. Waist circumference (WC) was measured halfway between the iliac crest and the bottom of the 12th costal bone, at the end of normal expiration and expressed in cm. Waist-to-height ratio (WHtR) was defined as waist circumference divided by height, both measured in the same units. Body weight was determined using Tanita Body Composition Analyzer (TBF-300MA, Tanita Corporation, Japan). Body mass index (BMI) was defined as the weight in kilograms divided by the square of the height in meters (kg/m^2^). The measurement of blood pressure was performed by using the Omron M3W, an electronic digital device for measuring blood pressure in the arm while the patient was seated in a chair.

### Questionnaire

In addition, men were asked to complete a questionnaire to provide information on medical and reproductive history and lifestyle factors including alcohol and smoking history. Drinking habits were screened using the AUDIT self-reported questionnaire [13]. A unit of alcohol was defined as 10 grams of pure alcohol.

### Semen analysis

Semen quality parameters like semen volume, total sperm count, sperm concentration, morphology and motility were assessed. In brief, semen samples were obtained by patient masturbation and all semen values were determined in accordance with WHO guidelines valid at the time of recruitment [10]. Leukocytospermia was defined according to WHO, definition for the neutrophil count >1million/ml.

A description of the standardized procedure used for semen analysis is given in detail elsewhere [12].

### Blood sampling

Blood samples were taken between 8 a.m. and 10.30 a.m. after an overnight fasting, on the same day that the semen sample was produced. Blood was centrifuged and serum was used to determine testosterone, oestradiol, follicle-stimulating hormone (FSH), luteinizing hormone (LH) and insulin concentrations by electrochemiluminescence immunoassay (ECLIA) method (Cobas e 601, Roche Diagnostics). Hexokinase method was used for serum glucose and enzymatic colorimetric method was used for HDL cholesterol and triglycerides assays (Cobas c 501, Roche Diagnostics).

### Statistical analysis

To obtain information on reproductive, anthropometrical and lifestyle findings FM and MPIC were divided into two groups according to the presence or non-presence of MS (MS^+^ or MS^-^, respectively). MS was defined according to the 2004 updated National Cholesterol Education Program (NCEP) Expert Panel on Detection, Evaluation, and Treatment of High Blood Cholesterol in Adults (Adult Treatment Panel III) (ATP III) criteria (at least three of the following criteria): waist circumference ≥ 102 cm; triglycerides ≥1.7 mmol/L (150 mg/dL) or drug treatment for elevated triglycerides; HDL less than 1.03 mmol/L (40 mg/dL); blood pressure ≥130/85 mm/Hg or use of medication for hypertension; fasting glucose ≥ 5.6 mmol/L (100 mg/dL) or use of medication for hyperglycemia [14].

Histograms and the P-P (probability-probability) plots were used to test for normality. Data regarding semen volume, sperm concentration and total sperm count were positively skewed. Skewed data were normalized by the natural log-transformation before analysis. The back-transformed adjusted mean values ensured the most reliable results and were used for evaluation below.

The independent t-test was applied to test for significant differences between the groups and the various parameters (anthropometric, lifestyle, reproductive parameters), with p < 0.05 considered statistically significant. To see whether distributions of categorical variables differ from each another, Pearson chi-square test was used. To explore the association between MS, semen quality and reproductive hormones, four groups of men were compared: FM-MS^-^, FM-MS^+^, MPIC-MS^-^, MPIC-MS^+^. To investigate the association between MS and reproductive parameters within BMI categories, these study participants were stratified into three standard BMI groups: (I) <25 kg/m^2^, (II) 25–29.9 kg/m^2^, (III) ≥30 kg/m^2^. The analysis was repeated. When determining the differences in reproductive parameters according to MS, we also took other known potential confounding factors into account using analyses of covariance (ANCOVA). We controlled for subject’s age, smoking, alcohol consumption and TTV. Missing information on smoking status and alcohol consumption restricted our data set (Table 1). We compared two groups of men (with whole data and with missing values) using t-test. We did not find any significant differences comparing data concerning age, testicular volumes, reproductive hormones and semen parameters.

**Table 1:** General characteristics of study groups.

	FM	MPIC	P
	(n = 238)	(n = 2642)	
**Personal characteristics, mean ± SD**			
Age in year	32.0 (6.1)	32.6 (5.7)	.118
Height (cm)	180.7(6.1)	181.3(6.9)	.155
BMI (kg/m^2^)	25.8 (3.8)	26.6 (4.5)	**.007** ${Χ}^{2}$
<25 n (%)	119 (50)	1047 (39.6)	
≥25<30 n (%)	83 (34.9)	1069 (40.5)	
≥30 n (%)	36 (15.1)	526 (19.9)	
Waist circumference (cm)	91.0 (9.7)	94.3 (12.4)	**< .001**
Waist-to-height ratio	0.504 (0.056)	0.521 (0.067)	**< .001**
**Reproductive characteristics, mean ± SD**			
Total testicular volume (ml)	48.0 (9.2)	45.8 (9.4)	**< .001**
Abstinent time (day)	3.8(1.6)	3.8(1.6)	.580
Semen volume (mL)	3.8 (1.6)	3.8 (2.1)	.831
Sperm concentration (×10^6^/mL)	74.6 (50.5)	38.6 (36.2)	**< .001**
Total sperm count (×10^6^)	282.3 (211.3)	144.0 (138.9)	**< .001**
Motile spermatozoa (%)	51.6(12.0)	41.4(16.5)	**< .001**
Normal morphology (%)	11.0 (5.6)	7.0 (5.3)	**< .001**
Serum FSH (IU/L)	4.0 (2.1)	4.4 (3.2)	**.027**
Serum LH (IU/L)	3.7 (1.8)	3.7 (1.9)	.991
Testosterone (nmol/L)	16.7(5.9)	16.7(6.1)	.918
Oestradiol (pmol/L)	123.6 (49.9)	127.6 (52.8)	.264
**Health and lifestyle characteristics, n (%)**			
MS	29(12.2)	471(17.8)	**.028** ${Χ}^{2}$
Testicular disorders ^a^	41(17.2)	498(18.8)	.539 χ2
Leukocytospermia	13(5.5)	238(9)	.063 χ2
Smoking status ^b^			**< .001** ${Χ}^{2}$
Never smoker	96(42.1)	427(21.9)	
Former smoker	66(28.9)	614(31.5)	
Current smoker	66(28.9)	909(46.6)	
Alcohol status ^c^			**.041** ${Χ}^{2}$
Non alkohol users	41(18.0)	225(12.1)	**.018** ${Χ}^{2}$
< 16 units/week	148(64.9)	1304(70.0)	
≥ 16 units/week	39(17.1)	333(17.9)	
Units per week, mean± SD	7.8(8.2)	8.8(11.1)	.212

**a** disorders affecting testicular function: varicocele (gr 2, 3 and bilateral), testicular

trauma, inguinal hernia

**b** FM: 10 missing, MPIC: 692 missing

**c** FM 10 missing

MPIC: 780 missing

The results are presented as mean (95% confidence intervals [CIs]) and as adjusted back-transformed mean values (95% CIs). Data analyses were performed using the SPSS for Windows version 20.0 (SPSS Inc. Chicago, IL). Statistical significance was defined at p < 0.05.

## Results

All the recruited men were Caucasian. The mean (SD) age of FM and MPIC was 32.0 (6.1) and 32.6 (5.7) years, respectively. Overall, 12.2% of FM and 17.8% of MPIC had MS according to the NCEP-ATP III criteria for MS [14]. General characteristics of FM and MPIC are shown in Table 1. When stratifying FM and MPIC according to the presence or non-presence of MS, men with MS in both groups were centrally obese as indicated by increased WC, WHtR and BMI (Table 2). Still, in the presence of MS, 3.4% of the FM and 6.8% of the MPIC were categorized as normal-weight (BMI <25 kg/m^2^). As expected, men in the MS group were found to have significantly higher triglycerides, systolic and diastolic blood pressure and decreased HDL cholesterol values as compared to men without MS in both groups. However, glucose concentrations were significantly higher only in the MPIC-MS^+^ group as well as fasting insulin levels (data available only for MPIC).

**Table 2:** Characteristics of FM and MPIC according to the presence or non-presence of MS.

	FM			MPIC		
	no MS	MS	P	no MS	MS	P
	(n = 209)	(n = 29)		(n = 2171)	(n = 471)	
**Personal characteristics, mean ± SD**						
Age in year	31.8(6.0)	33.4(6.7)	**.188**	32.1(5.6)	34.9(5.8)	**< .001**
Height (cm)	180.6(6.2)	180.9(5.5)	**.860**	181.2(7.0)	181.9(6.7)	.052
Total testicular volume (ml)	48.1(9.6)	47.1(8.0)	**.581**	45.3(8.8)	47.8(10.7)	**< .001**
Abstinent time (day)	3.8(1.5)	3.8(1.7)	**.844**	3.8(1.6)	3.9(1.8)	**.221**
**Anthropometric and MS characteristics, mean ± SD**						
BMI (kg/m^2^)	25.0(3.2)	31.4(3.7)	**< .001χ2**	25.6(3.8)	31.1(4.6)	**< .001 χ2**
<25 n (%)	118(56.5)	1(3.4)		1015(46.8)	32(6.8)	
≥25<30 n (%)	75(35.9)	8(27.6)		902(41.5)	167(35.5)	
≥30 n (%)	16(7.7)	20(69.0)		254(11.7)	272(57.7)	
Waist-to-height ratio	0.493(0.048)	0.582(0.045)	**< .001**	0.506(0.058)	0.589(0.066)	**< .001**
Waist circumference (cm)	89.1(8.2)	105.3(7.9)	**< .001**	91.6(10.6)	107.2(12.1)	**< .001**
Blood pressure (mm/Hg)						
Systolic BP	132.0(16.2)	141.6(16.7)	**.004**	132.0(16.3)	144.0(15.9)	**< .001**
Diastolic BP	78.4(9.5)	90.0(13.1)	**< .001**	79.9(10.0)	90.3(10.7)	**< .001**
HDLcholesterol (mmol/L)	1.4(0.3)	1.0(0.3)	**< .001**	1.4(0.3)	1.1(0.3)	**< .001**
Triglycerides (mmol/L)	1.2(0.6)	2.6(1.1)	**< .001**	1.2(0.7)	3.0(3.1)	**< .001**
Fasting glucose (mmol/L) ()l(L%vated BP (BP ≥130/85 mmHg or therapy) Central obesity (waist circumference >102 cm) Reduced HDL cholesterol (<40 mg/dL) or therapy Elevated triglycerides (≥150 mg/dL) or therapy Elevated fasting glucose (≥100 mg/dL)	5.0(0.6)	5.1(0.5)	**.251**	5.1(0.7)	5.7(1.6)	**< .001**
Fasting insulin (mU/L)				6.6(5.2)	11.9(7.4)	**< .001**
**Health and lifestyle characteristics, n (%)**						
Testicular disorders^a^	35(16.7)	6(20.7)	**.598 χ2**	416(19.2)	82(17.4)	**.378 χ2**
Leukocytospermia	11(5.3)	2(6.9)	**.717 χ2**	187(8.6)	51(10.8)	**.128 χ2**
Smoking status^b^			.060 χ2			**< .001 χ2**
Never smoker	90(44.8)	6(22.2)		373(23.5)	54(14.8)	
Former smoker	54(26.9)	12(44.4)		504(31.8)	110(30.2)	
Current smoker	57(28.4)	9(33.3)		709(44.7)	200(54.9)	
Alcohol status^c^			**.009χ2**			**.012 χ2**
Non alkohol users	39(19.4)	2(7.4)		193(12.6)	32(9.8)	
< 16 units/week	133(66.2)	15(55.6)		1087(70.7)	217(66.8)	
≥ 16 units/week	29(14.4)	10(37.0)		257(16.7)	76(23.4)	
Alcohol intake, units/week, mean ± SD						
	7.3(7.7)	11.5(10.9)	**.012**	8.4(11.0)	10.2(11.8)	**.011**

**a** disorders affecting testicular function: varicocele (gr 2, 3 and bilateral), testicular trauma, inguinal hernia

**b** FM: 10 missing, MPIC: 692 missing

**c** FM 10 missing, MPIC: 780 missing

Men with MS were older although a significant correlation was seen only in the MPIC group. Smoking and alcohol consumption were higher among men with MS. At the same time, disorders potentially affecting testicular function and leucocytospermia were not related to MS.

### MS and reproductive parameters

Significantly lower semen quality (except for semen volume) and TTV were seen in MPIC (Table 1). On the contrary, MS was positively correlated to the TTV seen in MPIC (Table 2). When stratifying men into four groups, the post hoc test showed the same results as mentioned above: significant differences in semen quality were only obtained between FM and MPIC groups (also seen within the BMI categories). At the same time, there were no significant correlations between FM-MS^-^ and FM-MS^+^ or MPIC-MS^-^ and MPIC-MS^+^ groups (Table 3). Likewise, no significant differences were found between MS and semen parameters within the BMI categories (S1 Table).

**Table 3:** Distribution of semen and hormonal parameters according to the presence or non-presence of MS.

	FM-MS ^-^	FM -MS ^+^	MPIC-MS ^-^	MPIC-MS ^+^	P
	(n = 209)	(n = 29)	(n = 2171)	(n = 471)	mean
**Semen volume (mL)**					
AMean(95%CI)	3.7(3.5; 4.0)	4 4.2(3.5; 5.0)	3.9(3.8; 4.0)	3.6(3.5; 3.8)	.052
**Sperm concentration** (**×10**^**6**^**/mL)**					
AMean(95%CI)	77.7(69.9; 86.3)	55.9(41.4; 75.4)	37.8(35.8; 40.0)	40.0(36.5; 45.9)	**< .001**^d^^,^ ^e^
**Total sperm count (×10**^**6**^**)**					
AMean(95%CI)	289.5(257.8;325.1)	234.9(175.2;314.5)	144.3(136.3;152.6)	142.9(126.1;162.1)	**< .001** ^d^^,^ ^e^
**Motile spermatozoa (%)**					
AMean(95%CI)	51.5(49.9; 53.2)	51.8(48.3; 55.4)	41.5(40.8; 42.1)	40.9(39.4; 42.4)	**< .001** ^d^^,^ ^e^^,^ ^f^^,^ ^g^
**Normal morphology (%)**					
Mean(95%CI)	11.2(10.5; 12.0)	9.2(7.3; 11.2)	7.0(6.7; 7.2)	6.9(6.4; 7.4)	**< .001** ^d^^,^ ^e^
**Serum FSH (IU/L)**					
Mean(95%CI)	4.2(3.8; 4.6)	4.0(3.3; 5.1)	4.3(4.2; 4.5)	4.3(4.0; 4.6)	.917
**Serum LH (IU/L)**					
Mean(95%CI)	3.7(3.4; 3.9)	4.2(3.6; 4.9)	3.6(3.5; 3.7)	3.3(3.1; 3.5)	**.004** ^c^
**Testosterone (nmol/L)**					
Mean(95%CI)	17.3(16.5; 18.1)	13.5(11.4; 15.7)	17.4(17.2; 17.7)	13.2(12.5; 13.8)	**< .001** ^a^^,^ ^b^^,^ ^e^
**Oestradiol (pmol/L)**					
Mean(95%CI)	124.0(117.2;130.9)	120.8(101.0;140.4)	130.6(127.7; 133.5) 134.4(129.0; 139.9)	139.6(133.5; 145.7)	**.007** ^e^

Reproductive parameters were adjusted for study age, alcohol use, smoking and TTV.

**a** Post-hoc pairwise comparisons with Bonferroni correction revealed P <0.001 FM-MS—versus FM-MS^**+**^

**b** Post-hoc pairwise comparisons with Bonferroni correction revealed P <0.001 MPIC-MS—versus MPIC-MS^**+**^

**c** Post-hoc pairwise comparisons with Bonferroni correction revealed P <0.05 MPIC-MS—versus MPIC-MS^**+**^

**d** Post-hoc pairwise comparisons with Bonferroni correction revealed P <0.01 FM-MS—versus MPIC-MS^**-**^

**e** Post-hoc pairwise comparisons with Bonferroni correction revealed P <0.01 FM-MS—versus MPIC-MS^**+**^

**f** Post-hoc pairwise comparisons with Bonferroni correction revealed P <0.01 FM-MS ^**+**^ versus MPIC- MS^**-**^

**g** Post-hoc pairwise comparisons with Bonferroni correction revealed P <0.01 FM-MS ^**+**^ versus MPIC- MS^**+**^

Regarding reproductive hormones, mean testosterone levels did not differ between the two groups, while testosterone was strongly correlated to MS (Table 3). This negative association persisted even after an adjustment for covariates within the BMI categories, but only between MPIC-MS^-^ and MPIC-MS^+^ groups (post hoc test p < 0.001 for BMI 25–29.9). In MPIC, LH was negatively correlated to MS, although the post hoc test did not show significant differences within the BMI categories. We found higher mean FSH levels in MPIC, but FSH as well as oestradiol levels were not related to MS.

## Discussion

To the best of our knowledge, this study was the first to report a significantly higher prevalence of MS in MPIC compared to FM of the same age range. Our findings showed that 17.8% of MPIC presenting for couples’ infertility met the criteria for MS of which the rate is higher than in previously reported findings [2, 3]. The difference between studies may come from the used definition and the prevalence of MS differing across countries as shown by Scuteri et al., (2015) [15]. We chose the adapted NCEP-ATP III criteria to define MS because this definition does not require insulin resistance or central obesity as a necessary diagnostic component and is widely used. Irrespective of the definition MPIC with MS seem to be older [2, 3], which was confirmed by our results.

Even if the prevalence of MS has been suggested as a possible risk factor that can attribute to infertility, the evidence linking MS with impaired semen parameters is still conflicting. Some of the studies, among them ours, indicate that MS did not seem to have a negative impact on semen parameters [3, 9]. Furthermore, when analysing the association between MS and semen quality by grouping the BMI into three categories, our results remained unchanged. We do not think that small number of participants in FM-MS^+^ (BMI <25 and BMI 25–29.9) groups have a considerable effect on results obtained. Conversely, other studies have found decreased semen volume [4], sperm concentration [4, 7] and decline in percentage of sperm with normal morphology [2, 4, 8] in men with MS. Nevertheless, negative correlation between semen parameters and MS from the studies mentioned above was obtained from MPIC, infertile men and men from a specific region of South Africa who were older compared to our study groups.

Apparently there are other undefined and potentially more harmful factors impairing semen quality apart from MS, especially in the case of MPIC. Leukocytospermia and testicular disorders may be associated with infection and poor sperm quality. However, we did not find any significant differences between the results of FM and MPIC as well as in the case of results of men with and without MS in both groups. Moreover, Leisegang et al. (2014) suggested that in the case of MS reduced fertility potential reveals also in the absence of leucocytospermia and clinical detection of varicocoele. It could be that a combination of certain components of MS and/or the combination of MS with some other factors plays a role.

Although we failed to show an independent relationship between semen quality and MS within BMI categories, based on our preliminary findings, differences in glycose and insulin levels seem to be interesting in the context of MS and reproductive health. Significantly higher glucose levels were found in MPIC-MS^+^ but not in FM*-*MS^+^ compared to men without MS. Besides, higher insulin levels were found in MPIC-MS^+^. Pitteloud et al. (2005) suggested that Leydig cell steroidogenesis is impaired in insulin-resistant states such as obesity and Leisegang et al. (2014) proposed that hyperinsulinemia is associated with increased seminal insulin concentrations, which may negatively impact the male reproductive function in the case of obesity [16; 7].

In regard to the hormones, we found that testosterone levels were negatively associated with MS, which also appeared within BMI categories but only between MPIC-MS^-^ and MPIC-MS^+^ groups. There is compelling evidence that low testosterone levels are not only one of the adverse consequences of MS but can be also a risk factor [17]. A negative association between testosterone levels and MS have been confirmed by many researches [2–5, 17] and a systematic review with meta-analysis showed that MS is significantly and independently associated with an overall lower total testosterone levels [18]. Concurrently, oestrogens were not related to MS. As expected, a higher level of FSH, a common indicator of spermatogenesis impairment, were seen in MPIC compared to FM. Central obesity and MS associated lower testosterone levels could also have been compensated by increased levels of gonadotropins, but this was not observed. Results from the European Male Aging Study supported the hypothesis according to which obesity associated hypothalamic-pituitary dysregulation blunts gonadotropins rise which cannot be compensated by physiological mechanisms [19,20]. Moreover, in our study we found lower levels of LH seen in MPIC-MS^+^ compared to MPIC-MS^—^group. In spite of statistically negative results, the same trend was seen within BMI groups. As already demonstrated before [21] lower LH levels could be explained by larger testicles found in MPIC with MS. Furthermore, glucose and insulin metabolism are believed to influence gonadotropins. Previous studies have shown that low testosterone levels in diabetic men are associated with low serum LH [22, 23]. Costanzo et al., (2014) hypothesized that impairment of hypothalamic activity appears in diabetic because of the inhibitory effect of hyperglycemia and insulin resistance [24]. Chosich et al., (2017) recently showed that hyperinsulinemia combined with elevated lipids suppresses LH and FSH and neither lipids nor insulin have this effect on their own [25].

There is also increasing evidence that lifestyle factors such as alcohol consumption [26–30] and smoking [31–35] affect fertility. Based on the previously mentioned outcomes, decline in male reproductive health, especially in MPIC with MS, can be explained, at least in part, by smoking and drinking habits as the presence of MS was associated with a less healthy lifestyle. However, significant changes in reproductive parameters persisted even after an adjustment for smoking and drinking.

A major **limitation** of our research can be the lack of SHBG for calculation because it is well known that obesity and MS are associated with the reduction of SHBG levels. Also, fasting serum insulin levels were available only from MPIC and our ANCOVA analysis was restricted by missing information regarding lifestyle parameters.

**In conclusion,** this study showed a significantly higher prevalence rate of MS in MPIC compared to FM. Beside testosterone levels, which were influenced by MS, the obtained results suggest that MS appears to have no independent impact on semen quality. Whether the combination of certain components of MS affects reproductive health or some other factors play a causal role, irrespective of the presence of MS, looking for their links to infertility remains to be elucidated.

## Supporting information

S1 Table Distribution of semen and hormonal parameters according to the presence or non-presence of MS within BMI categories. (DOC)
