# Supplementary material for: The effect of metabolic syndrome on male reproductive health: A cross-sectional study in a group of fertile men and male partners of infertile couples
Source: PLoS One. 2018 Mar 16;13(3):e0194395. doi: 10.1371/journal.pone.0194395 (PMC5856494; doi:10.1371/journal.pone.0194395)
Supplement: S1 Table — (DOC) [file pone.0194395.s001.doc]

**Supplemental table S1. Distribution of** semen and hormonal parameters according to the presence or non-presence of MS within BMI categories.

|  | **Semen volume**  (mL) | **Sperm**  **concentration**  (×10^6^/mL) | **Total sperm count**  (×10^6^) | | **Motile**  **spermatozoa**  (%) | **Normal morphology** (%) | **Serum FSH**  (IU/L) | **Serum LH**  (IU/L) | **Testosterone**  (nmol/L) | **Oestradiol**  (pmol/L) |
| --- | --- | --- | --- | --- | --- | --- | --- | --- | --- | --- |
| **BMI <25** |  |  |  | |  |  |  |  |  |  |
| **FM-MS^-^**  (n=118) | 3.8(3.5; 4.1) | 74.2(60.9; 90.5) | 281.7(229.5; 346.2) | | 49.6(46.6; 52.6) | 10.5(9.5; 11.5) | 4.3(3.9; 4.8) | 3.9(3.6; 4.2) | 19.1(17.9; 20.2) | 121.0(111.2; 130.8) |
| **FM -MS^+^**  (n=1) | 7.9 | 24.5 | 193.7 | | 42.4 | 3.0 | 4.6 | 4.0 | 11.0 | 118.0 |
| **MPIC-MS^-^** (n=1015) | 3.8(3.7; 4.0) | 39.4(36.3; 42.9) | 153.2(140.6; 167.2) | | 41.1(39.8; 42.3) | 6.9(6.5; 7.3) | 4.2(4.0; 4.4) | 3.6(3.5; 3.8) | 19.1(18.6; 19.6) | 128.4(124.2; 132.6) |
| **MPIC-MS^+^**  (n=32) | 3.4(2.8; 4.0) | 46.3(29.7; 72.0) | 155.6(98.2; 246.4) | | 40.8(34.0; 47.6) | 8.9(6.7; 11.2) | 3.9(2.9; 5.0) | 3.0(2.3; 3.7) | 16.3(13.7; 18.8) | 153.1(131.3; 175.0) |
| **p value** | .145 | <.001 | <.001 | | <.001 | <.001 | .899 | .127 | .129 | .073 |
| **BMI 25-29.9** |  |  |  | |  |  |  |  |  |  |
| **FM-MS^-^**  (n=75) | 3.8(3.5; 4.2) | 59.9(45.8; 78.5) | 227.9(171.1; 303.4) | | 51.8(48.1; 55.6) | 11.9(10.7; 13.2) | 3.9(3.2; 4.6) | 3.5(3.1; 4.0) | 15.5(14.3; 16.6) | 129.1(117.1; 141.1) |
| **FM -MS^+^**  (n=8) | 5.3(4.0; 7.2) | 41.8(18.7; 93.5) | 222.1(94.3; 522.7) | | 53.4(42.2; 64.6) | 7.3(3.6; 11.0) | 4.0(1.9; 6.0) | 3.8(2.5; 5.1) | 15.4(11.9; 18.9) | 117.3(81.5; 153.1) |
| **MPIC-MS^-^** (n=902) | 3.8(3.7; 4.0) | 38.9(35.2; 42.9) | 147.1(132.4; 163.2) | | 40.4(39.0; 41.7) | 7.4(6.9; 7.8) | 4.5(4.2; 4.7) | 3.5(3.4; 3.7) | 16.3(15.9; 16.8) | 132.6(128.2; 137.0) |
| **MPIC-MS^+^**  (n=167) | 3.8(3.5; 4.1) | 42.4(33.9; 53.0) | 152.2(120.1; 192.9) | | 39.7(36.6; 42.8) | 7.1(6.1; 8.1) | 4.4(3.9; 5.0) | 3.1(2.8; 3.5) | 13.1(12.1; 14.1) | 136.7(126.7; 146.7) |
| **p value** | .186 | .032 | .035 | | <.001 | <.001 | .553 | .230 | <.001 | .650 |
| **BMI ≥ 30** |  |  |  | |  |  |  |  |  |  |
| **FM-MS^-^**  (n=16) | 2.6(2.0; 3.2) | 76.3(39.7; 146.5) | 194.8(101.9; 372.8) | | 56.4(47.8; 65.0) | 10.9(8.2; 13.5) | 4.5(3.0; 6.0) | 3.6(2.6; 4.5) | 12.5(10.1; 14.9) | 134.9(108.0; 161.8) |
| **FM -MS^+^**  (n=20) | 3.6(3.0; 4.5) | 57.6(31.7; 104.6) | 211.2(116.6; 382.2) | | 51.2(43.4; 59.1) | 9.6(7.1; 12.0) | 4.0(2.6; 5.3) | 4.5(3.6; 5.3) | 12.5(10.3; 14.6) | 123.6(99.0; 148.1) |
| **MPIC-MS^-^** (n=254) | 3.9(3.7; 4.2) | 33.3(27.2; 40.7) | 131.6(107.7; 160.9) | | 39.7(37.0; 42.4) | 6.7(5.8; 7.5) | 4.5(4.1; 5.0) | 3.7(3.4; 4.0) | 13.8(13.0; 14.5) | 130.0(121.4; 138.6) |
| **MPIC-MS^+^**  (n=272) | 3.6(3.0; 3.8) | 38.2(31.5; 46.4) | 138.0(113.8; 167.3) | | 41.6(39.0; 44.1) | 7.0(6.3; 7.9) | 4.2(3.7; 4.6) | 3.4(3.1; 3.7) | 12.8(12.1; 13.5) | 140.8(132.5; 149.0) |
| **p value** | .002 | .05 | .358 | | <.001 | .006 | .586 | .090 | .216 | .250 |
|  |  |  |  |  | |  |  |  |  |  |
|  | | | | | | |  |  |  |  |

Reproductive parameters were adjusted for study age, alcohol use, smoking and TTV.
